# Numerical Stability Analysis of an Acoustically Levitated Thin Reflective Plate for Contactless Optical Beam Steering

**DOI:** 10.3390/mi17080879

**Published:** 2026-07-24

**Authors:** Zhao Liu, Hu Yang, Haotong Ma

**Affiliations:** 1 State Key Laboratory of Optical Field Manipulation Science and Technology, Institute of Optics and Electronics, Chinese Academy of Sciences, Chengdu 610209, China; tracyliuzhao1@163.com (Z.L.); yangh@ioe.ac.cn (H.Y.); 2 Key Laboratory of Optical Engineering, Chinese Academy of Sciences, Chengdu 610209, China

**Keywords:** acoustic levitation, contactless optical beam steering, thin reflective plate, acoustic radiation force, Gor’kov potential, stability analysis

## Abstract

Acoustically levitated thin reflective plates provide a promising approach for contactless optical beam steering by eliminating mechanical hinges, sliding interfaces, and the associated wear and friction losses. However, unlike conventional spherical particles or droplets, thin planar reflectors exhibit highly geometry-dependent acoustic force distributions and are highly susceptible to lateral drift and angular destabilization when the acoustic field is dynamically reconfigured for beam steering. Here, we present a theoretical and simulation-based stability analysis of an acoustically levitated thin reflective plate driven by a phase-controlled dual-array acoustic field. A reduced-order model based on the Gor’kov potential is developed to characterize the acoustic potential landscape, escape-barrier depth, and local restoring stiffness during phase-gradient-induced mirror tilting. The simulations reveal that increasing the phase gradient progressively distorts the trapping potential and reduces the available trapping stability margin. Among the translational degrees of freedom, the lateral restoring stiffness deteriorates much more rapidly than the axial stiffness, indicating that lateral slippage is the primary instability pathway during continuous steering. Parametric analysis further shows that thinner mirrors with larger radii can improve trapping stability by increasing the effective acoustic interaction area while reducing gravitational and inertial penalties. The influence of non-ideal acoustic driving conditions is also evaluated to determine practical operating limits for stable operation. These results clarify the stability mechanisms governing acoustically suspended planar reflectors and provide theoretical design guidelines for robust contactless optical beam-steering systems.

## 1. Introduction

Optical beam steering is an important technology in applications such as light detection and ranging (LiDAR), free-space optical communications, and adaptive optics. Traditional mechanical beam-steering mechanisms, such as galvanometric or polygonal scanners, offer large clear apertures but are limited by their physical size and mechanical wear in dynamic environments. Microelectromechanical systems (MEMS) micromirrors and solid-state optical phased arrays (OPAs) serve as compact alternatives [1,2,3]. However, MEMS micromirrors face trade-offs between aperture size and steering angle, as well as potential material fatigue [2]. Meanwhile, OPAs avoid mechanical movement but often encounter high insertion losses and complex fabrication requirements [4,5]. Therefore, alternative beam-steering mechanisms that maintain large apertures while eliminating mechanical friction are highly desirable.

Mid-air acoustic levitation offers a non-contact method for trapping and manipulating objects by utilizing the acoustic radiation force generated by phased transducer arrays [6,7,8,9]. Combining acoustic levitation with optical elements introduces the concept of a contactless reflective mirror. In our previous work [10], we demonstrated the feasibility of this mechanism by suspending a miniature mirror using counter-propagating ultrasonic fields. The initial prototype successfully achieved discrete optical beam steering through an acoustic switching-control scheme. This proof-of-concept demonstrated the feasibility of a hinge-free beam-steering architecture. However, that study primarily focused on demonstrating the optical deflection concept and did not address the underlying levitation stability of the mirror body when the acoustic field is manipulated.

Although acoustic levitation has been extensively investigated for particles, droplets, and bulk samples, most theoretical models and experimental demonstrations have focused on spherical or nearly spherical objects [11]. Recent reviews have also emphasized that particle shape and acoustic scattering can strongly modify the resulting radiation forces and dynamic behavior [11]. In contrast, studies on acoustically levitated planar or disk-like objects remain relatively limited. Hashimoto et al. demonstrated near-field acoustic levitation of planar specimens for noncontact transportation, showing that stable suspension depends strongly on the specimen size relative to the flexural vibration wavelength [12]. Hu et al. further analyzed the lateral stability of an acoustically levitated disk by considering acoustic viscous stress and introduced the stability coefficient and holding force to quantify the tendency of a disk to slip from equilibrium [13]. Xie and Wei showed that the acoustic radiation force on disk samples can deviate significantly from the classical spherical-particle scaling, and that thin disks may exhibit enhanced levitation force depending on their aspect ratio and radius [14]. These studies indicate that planar objects introduce geometry-dependent force distributions and stability constraints that are absent or less pronounced in conventional spherical-particle levitation. However, they mainly address near-field suspension, static disk levitation, or disk-like samples in fixed standing-wave fields. The stability of a freely suspended thin reflective plate under phase-controlled acoustic tilting, especially for contactless optical beam steering, has not yet been systematically clarified.

Maintaining stable levitation for a planar, non-spherical object like a thin reflective plate is inherently more challenging than levitating conventional spherical particles. When phase gradients are adjusted to tilt the mirror for beam steering, the distribution of acoustic radiation forces across the mirror surface shifts, directly altering the local acoustic-potential landscape. This structural alteration can easily compromise the levitation stability, potentially causing unpredictable angular oscillations, positional drift, or complete ejection of the mirror from the acoustic trap. Therefore, a comprehensive understanding of how the driving phases, mirror geometries, and acoustic conditions cooperatively dictate the levitation stability limits is essential to ensure the reliability of such contactless platforms.

To address this issue, this paper presents a dedicated theoretical and simulation-based analysis of the levitation stability of a thin reflective plate acoustically suspended in a phase-gradient controlled dual-array system. Using a reduced-order model based on the Gor’kov potential, we systematically evaluate the static and dynamic stability boundaries of the system. By quantitatively characterizing core stability metrics—specifically the potential escape-barrier depth and the localized effective restoring stiffnesses—we map the degradation of the acoustic trap during continuous steering operations.

The remainder of this manuscript is organized as follows. Section 2 introduces the physical configuration and stability metrics of the system, investigates the static potential-well variations induced by phase-gradient beam steering, and analyzes the effects of different mirror geometries on the levitation stability. Section 3 examines the influence of non-ideal acoustic driving conditions on these stability parameters and synthesizes these findings into practical operational guidelines. Finally, Section 4 summarizes the core conclusions of this study and notes the current modeling limitations.

## 2. Theoretical Framework and Static Stability Analysis

The stability analysis in this work combines two complementary methods. A reduced-order model based on the Gor’kov potential is used to efficiently explore the geometric and phase-gradient parameter space and to define the stability metrics (escape-barrier depth ΔU and restoring stiffnesses *k*_x_, *k*_z_). Full-wave finite-element simulation is then used at representative operating points to independently validate the key conclusions without invoking the long-wavelength approximation. The following subsections define the coordinate system, the stability metrics, and the equivalent dynamic model in turn.

**Figure 1:**
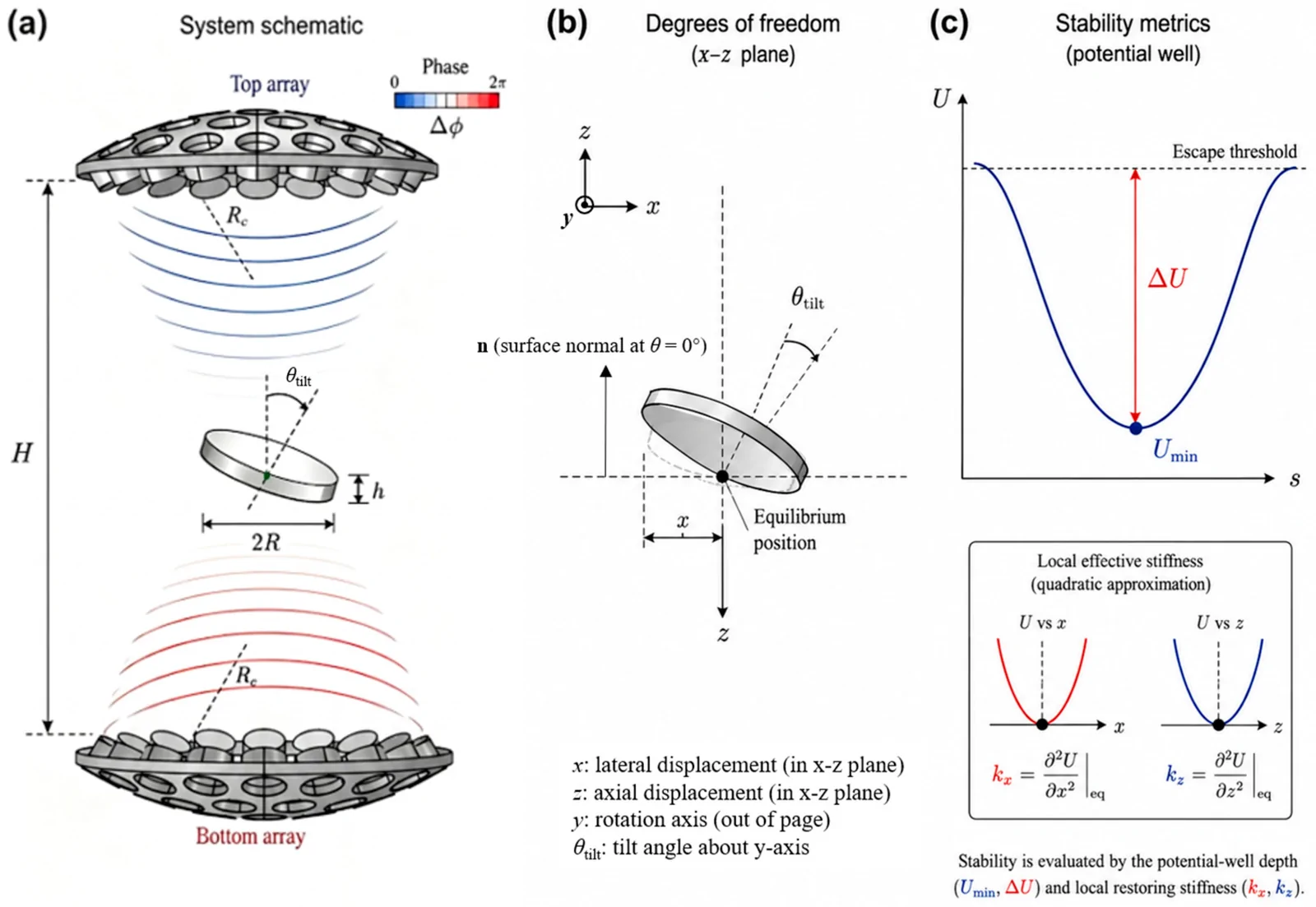
Structural schematic and metrics definition of the acoustically levitated thin reflective plate system: (**a**) System schematic of the contactless optical beam-steering framework actuated by opposed top and bottom transducer arrays with phase-shift controls; (**b**) Analytical coordinate system of the mirror in the *x*-*z* plane. The *y*-axis (out of page, marked ⊙) is the rotation axis; the mirror tilts by angle *θ*_tilt_ within the *x*-*z* plane. The surface normal ***n*** is along *z* at zero tilt. The translational degrees of freedom are the lateral displacement *x* and the axial displacement *z*; (**c**) Topography of the time-averaged acoustic-potential field *U*(x, z) delineating the local equilibrium center, the global escape-barrier depth ΔU, and the effective localized restoring stiffnesses *k*_x_ and *k*_z_.

To establish a quantitative foundation for evaluating the trapping security of the contactless beam-steering platform, the coordinate system, physical degrees of freedom, and the primary stability metrics must be strictly formulated. The analytical architecture of the dual-array acoustic levitation system is schematically illustrated in Figure 1.

The acoustic field is established by two opposed, phase-controlled transducer arrays operating in a counter-propagating manner. The miniature reflective thin plate is levitated in mid-air near the acoustic pressure nodes. Within the primary *x*-*z* plane, the spatial status of the mirror is defined by three independent degrees of freedom: the lateral displacement *x*, the axial displacement *z*, and the acoustic-induced tilt angle *θ*_tilt_.

The interaction between the ultrasonic field and the planar object is characterized by the time-averaged acoustic radiation forces, which are described here using a reduced-order formulation derived from the Gor’kov potential framework [15,16]. Because the mirror size is comparable to the acoustic wavelength, the present Gor’kov-potential-based formulation is used as an effective reduced-order model to evaluate relative stability trends, rather than as a full scattering solution for the exact acoustic force distribution. Specifically, at 40 kHz (*λ* ≈ 8.6 mm), the mirror diameter (2*R* ≈ 0.7*λ*) lies outside the strict long-wavelength regime in which the Gor’kov potential is formally derived. We therefore use it for parametric design exploration and the analytical description of translational trapping, and independently verify the key stability conclusions by full-wave finite-element simulation, which makes no long-wavelength approximation (Section 3). Under this approximation, the effective acoustic potential of the levitated thin reflective plate can be expressed as follows:

(1) $$U\left(x,z\right)=V_{eff}\left[\frac{1}{2}f_{1}\frac{\langle p{\left(x,z\right)}^{2}\rangle }{\rho_{0}c_{0}^{2}}-\frac{3}{4}f_{2}\rho_{0}\langle v{\left(x,z\right)}^{2}\rangle \right]$$

where *U*(*x*, *z*) is the effective acoustic potential, and *V_eff_* denotes an effective geometry-dependent acoustic interaction factor of the thin reflective plate. In the present reduced-order model, *V_eff_* is introduced to capture the increase in acoustic coupling with the plate radius and interaction area, rather than to represent the exact solid volume of the disk. The exact geometric volume of a thin disk scales as *πR*^2^*h*, while the effective acoustic interaction also depends on the projected area, characteristic length scale, and acoustic-field distribution over the plate surface. Therefore, *V_eff_* is used here as a reduced-order coupling parameter for evaluating relative stability trends. The quantities ⟨*p*(*x*, *z*)^2^⟩ and ⟨*v*(*x*, *z*)^2^⟩ are the time-averaged mean-square acoustic pressure and particle velocity, respectively. The parameters *ρ*_0_ and *c*_0_ are the density and speed of sound in air, and *f*_1_ and *f*_2_ are the acoustic contrast factors associated with the compressibility and density contrast between the levitated object and the surrounding medium. In the dual-array setup, the introduction of a phase gradient *ϕ* actively alters the synthesized pressure field *p*(*x*, *z*), which can be analytically approximated as follows:

(2) $$p\left(x,z\right)=2p_{0}\cos\left(kz+\frac{\phi x}{d}\right)$$

where *p*_0_ is the pressure amplitude of a single transducer, *k* is the wave number, and dis the characteristic array spacing. This fundamental coupling reveals how electrical phase shifts physically distort the local pressure nodes. The spatial distribution of this potential energy, denoted as *U*(*x*, *z*), forms a localized acoustic-potential landscape that maps the spatial equilibrium of the suspended mirror.

To rigorously evaluate the vulnerability of the mirror to external perturbations or steering-induced forces, two categories of quantitative stability metrics are mathematically defined. The first is the escape-barrier depth Δ*U*, which represents the global safety margin of the acoustic trap. It is defined as the potential energy increment required for the mirror to escape from its local equilibrium minimum (*x*_0_, *z*_0_) to the nearest potential barrier or saddle point:

(3) $$\Delta U=U_{saddle}-U\left(x_{0},z_{0}\right)$$

A deeper potential well implies that the system possesses a greater capacity to withstand aerodynamic drag, acoustic streaming, or inertial loads without experiencing complete particle ejection. The second category includes the effective restoring stiffnesses *k_x_* and *k_z_*, which govern the localized restoring performance. They are calculated via the second-order spatial derivatives of the acoustic potential energy evaluated at the equilibrium center:

(4) $$k_{x}=\frac{\partial^{2}U}{\partial x^{2}}\left|\left(x_{0},z_{0}\right)\right.,\;k_{z}=\frac{\partial^{2}U}{\partial z^{2}}\left|\left(x_{0},z_{0}\right)\right.$$

Furthermore, to assess the dynamic resilience of the levitated mirror against operational vibrations, the system can be modeled near its equilibrium state using linearized second-order dynamic equations. For lateral translation, this is expressed as *m*d^2^*x*/d*t*^2^ + *c*d*x/*d*t* + *k_x_x* = *F*_ext_ (*t*), where m is the mirror mass, cis the fluid viscous damping coefficient [17,18], and *F*_ext_ (*t*) is the external disturbance force. This dynamic framework enables the extraction of the fundamental natural restoring frequency *f_n_* and the system’s settling time *t_s_*:

(5) $$f_{n}=\frac{1}{2\pi }\sqrt{\frac{k_{eff}}{m}},\;t_{s}\approx \frac{4}{\zeta 2\pi f_{n}}$$

where *k_eff_* represents the corresponding effective stiffness and *ζ* is the damping ratio. These analytical expressions lay the theoretical groundwork, demonstrating that both the static trapping limits and dynamic responsiveness are intrinsically coupled to the geometric design (*V_eff_*, *m*) and acoustic field variations (*p*_0_, *ϕ*).

**Figure 2:**
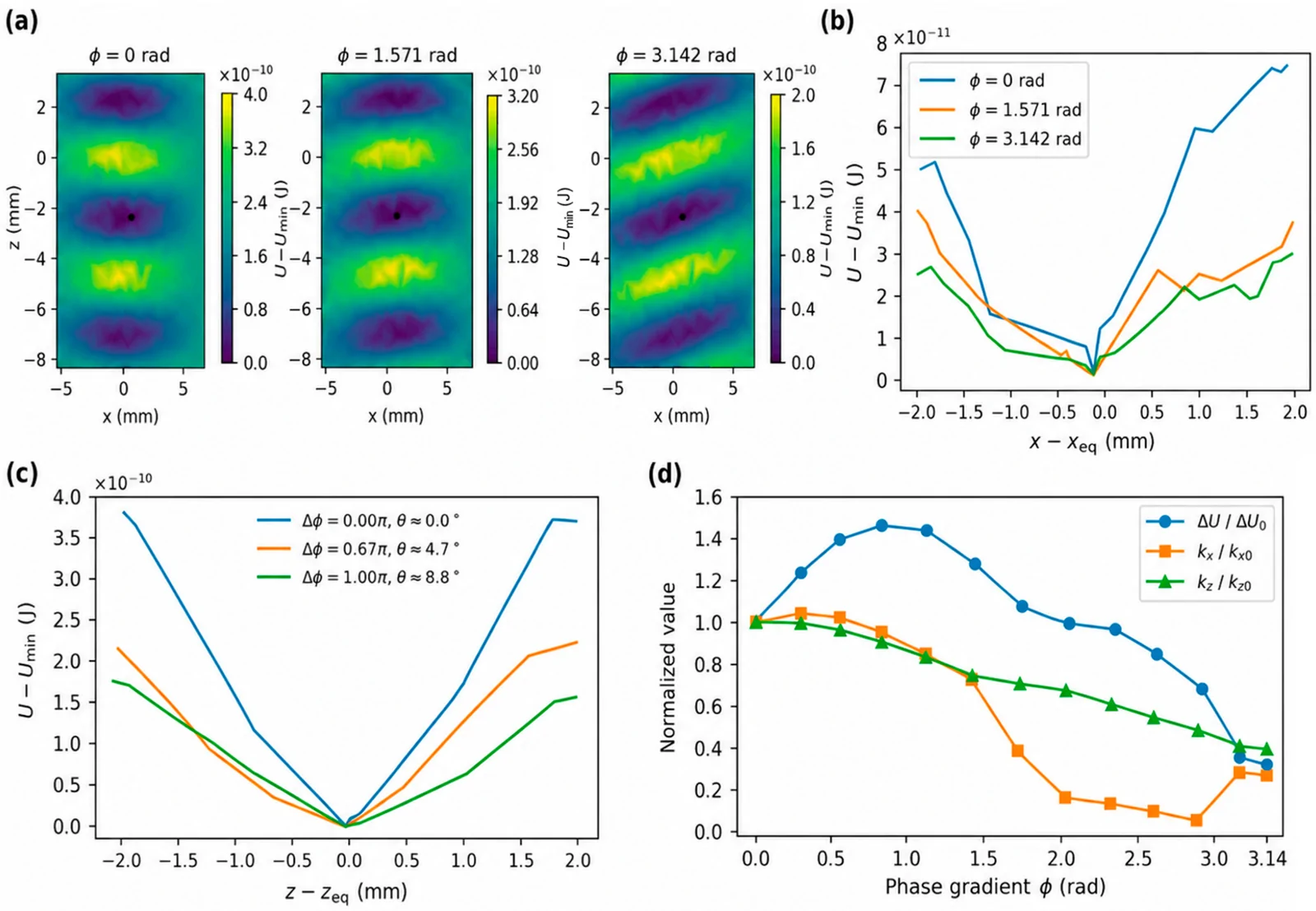
Evaluation of phase-gradient induced acoustic-potential well distortions: (**a**) Two-dimensional spatial profiles of the potential landscape *U*(*x*, *z*) under progressive phase gradients of 0 rad, *π*/2, and *π*; One-dimensional potential energy cuts extracted along (**b**) the horizontal lateral displacement and (**c**) the vertical axial displacement for different mirror tilt angles; (**d**) Normalized degradation curves of the core stability metrics across the full phase-gradient operating spectrum.

The equivalent mechanical model in Equation (5) is obtained under four assumptions. First, the thin plate is treated as a rigid body, valid because its first flexural resonance lies far above the acoustic and suspension frequencies, so no elastic deformation is excited. Second, the acoustic restoring force is linearized about the equilibrium point, where the acoustic potential forms a smooth single-minimum well (Figure 2b,c); the effective stiffness is *k*_eff_ = *d*^2^*U*/*dq*^2^|_0_ and the natural frequency is *f*_n_. Third, the translational and rotational degrees of freedom are treated as decoupled, as justified by the distinct evolution of their restoring metrics (Section 3). Fourth, dissipation is modeled as linear viscous damping following Refs. [17,18], giving an underdamped response consistent with the observed sustained oscillation. Each rigid-body degree of freedom obeys the same generic second-order form; Equation (5) is written for the lateral coordinate, which governs the stability limit, so that *f*_n_ characterizes the lateral dynamic stiffness at each steering angle.

When continuous phase shifts are applied to the transducer arrays to introduce a phase gradient *ϕ*, the acoustic standing-wave field undergoes the aforementioned spatial reconfiguration, tilting the acoustic potential strips to steer the mirror orientation. However, this steering mechanism introduces severe structural distortion to the local potential landscape. The numerical simulation results mapping these variations as a function of the phase gradient *ϕ* are presented in Figure 2.

In the initial unsteered state at 0 rad, the potential well exhibits a perfectly symmetric, horizontal strip profile with the global minimum centered precisely at the origin, achieving a maximum trap depth of 4.0 × 10^−10^ J. As the phase gradient is increased to π/2, the potential strips undergo a synchronized spatial inclination. Concurrently, the maximum potential depth drops to 3.2 × 10^−10^ J. When the phase gradient reaches its extreme state at π, the potential topography is severely degraded; the boundary barriers flatten significantly, and the peak potential depth is halved to 2.0 × 10^−10^ J. This transition clearly demonstrates that large-angle beam steering compromises the energy containment capability of the acoustic trap.

The localized effects of this potential distortion are further elucidated by the one-dimensional potential energy cuts. The lateral potential energy profile along the horizontal offset (*x* − *x*_eq_) gradually loses its parabolic symmetry as *ϕ* increases. The potential slope on the positive escape side becomes noticeably shallower, indicating a drastic reduction in the lateral confinement force. Similarly, the axial potential energy profiles show that as the mirror tilts from 0.0° to 8.8°, the potential valley flattens significantly, and the effective axial trap width narrows, meaning that the vertical positional stability of the mirror degrades continuously during angular rotation.

Tracking the normalized evolution of the stability metrics across the entire phase-gradient operating range provides a comprehensive overview of this degradation. The normalized trap depth Δ*U*/Δ*U*_0_ exhibits a non-monotonic trend, rising slightly to a peak of approximately 1.45 near 0.8 rad due to localized constructive interference, before suffering a monotonic, severe decline down to 0.3 at maximum gradient. Crucially, the normalized lateral stiffness *k_x_*/*k*_*x*0_ undergoes a sharp decrease past 1.0 rad, approaching near-zero values at the steering boundary. In contrast, the normalized axial stiffness *k_z_*/*k*_*z*0_ experiences a much more gradual and well-behaved linear decrease, retaining roughly 40% of its baseline performance at the maximum gradient. This divergence reveals that the primary failure mode of the levitated mirror during continuous phase steering is not vertical unseating, but rather lateral sliding or slippage caused by the acute loss of lateral restoring stiffness *k_x_*.

The full-wave simulation was performed in the Pressure Acoustics, Frequency Domain interface, solving the Helmholtz equation at 40 kHz in air (*ρ*_0_ = 1.2 kg/m^3^, *c*_0_ = 343 m/s). The field was generated by two opposed hemispherical arrays of 36 transducers each, modeled as normal-velocity boundaries; the mirror was a sound-hard boundary, the domain was terminated by perfectly matched layers, and the mesh used a maximum element size of *λ*/10. This full-wave formulation makes no long-wavelength (*r* ≪ *λ*) approximation and is valid at the actual mirror-to-wavelength ratio (2*R* ≈ 0.7*λ*, where *λ* ≈ 8.6 mm at 40 kHz).

The restoring torque about the tilt (*y*) axis was obtained by direct surface integration of the acoustic radiation stress over the mirror:

(6) $$T_{y}=\iint_{surface}\left[\left(x-x_{0}\right)\cdot P_{rad}\cdot n_{z}-\left(z-z_{0}\right)\cdot P_{rad}\cdot n_{x}\right]dS$$

where *P*_rad_ is the acoustic radiation pressure, (*x*_0_, *z*_0_) is the rotation center, and *n*_x_, *n*_z_ are the outward surface-normal components.

### 2.1. Full-Wave FEM Simulation

To validate the stability characteristics predicted by the reduced-order model, a three-dimensional full-wave acoustic simulation was performed using COMSOL Multiphysics 6.3. The Pressure Acoustics, Frequency Domain interface was employed to solve the Helmholtz equation for harmonic acoustic propagation at an operating frequency of 40 kHz. The surrounding medium was air with a density of *ρ* = 1.2 kg/m^3^ and a speed of sound of *c* = 343 m/s, corresponding to an acoustic wavelength of approximately *λ* = 8.6 mm.

The computational domain consisted of an air region containing two opposed hemispherical ultrasonic transducer arrays and a thin reflective plate positioned near the acoustic equilibrium region. Each hemispherical array consisted of 36 ultrasonic transducers. The transducer surfaces were modeled as normal velocity boundary conditions, and the phase excitation of each element was individually adjusted to generate the required phase-gradient acoustic field for mirror steering.

The levitated reflector was modeled as a circular aluminum plate with a radius of *R* = 3 mm and a thickness of *h* = 0.2 mm. The material density of the aluminum mirror was set to 2700 kg/m^3^. Since the plate thickness is much smaller than the acoustic wavelength, the mirror was treated as a rigid sound-hard boundary in the acoustic field simulation, with zero normal acoustic velocity at the reflective surface.

A perfectly matched layer (PML) was applied at the outer boundaries of the acoustic domain to eliminate artificial wave reflections. The computational mesh was generated using physics-controlled tetrahedral elements with local refinement around the transducer surfaces and the mirror boundary. The maximum mesh element size was limited to *λ*/10, providing sufficient spatial resolution for accurately capturing the acoustic pressure distribution and scattering characteristics around the reflective plate.

The acoustic radiation pressure acting on the mirror surface was extracted from the simulated acoustic field. The restoring torque around the tilt axis was calculated by integrating the acoustic radiation stress over the mirror surface. The resulting torque distribution was further integrated with respect to the tilt angle to obtain the rotational potential energy landscape.

**Figure 3:**
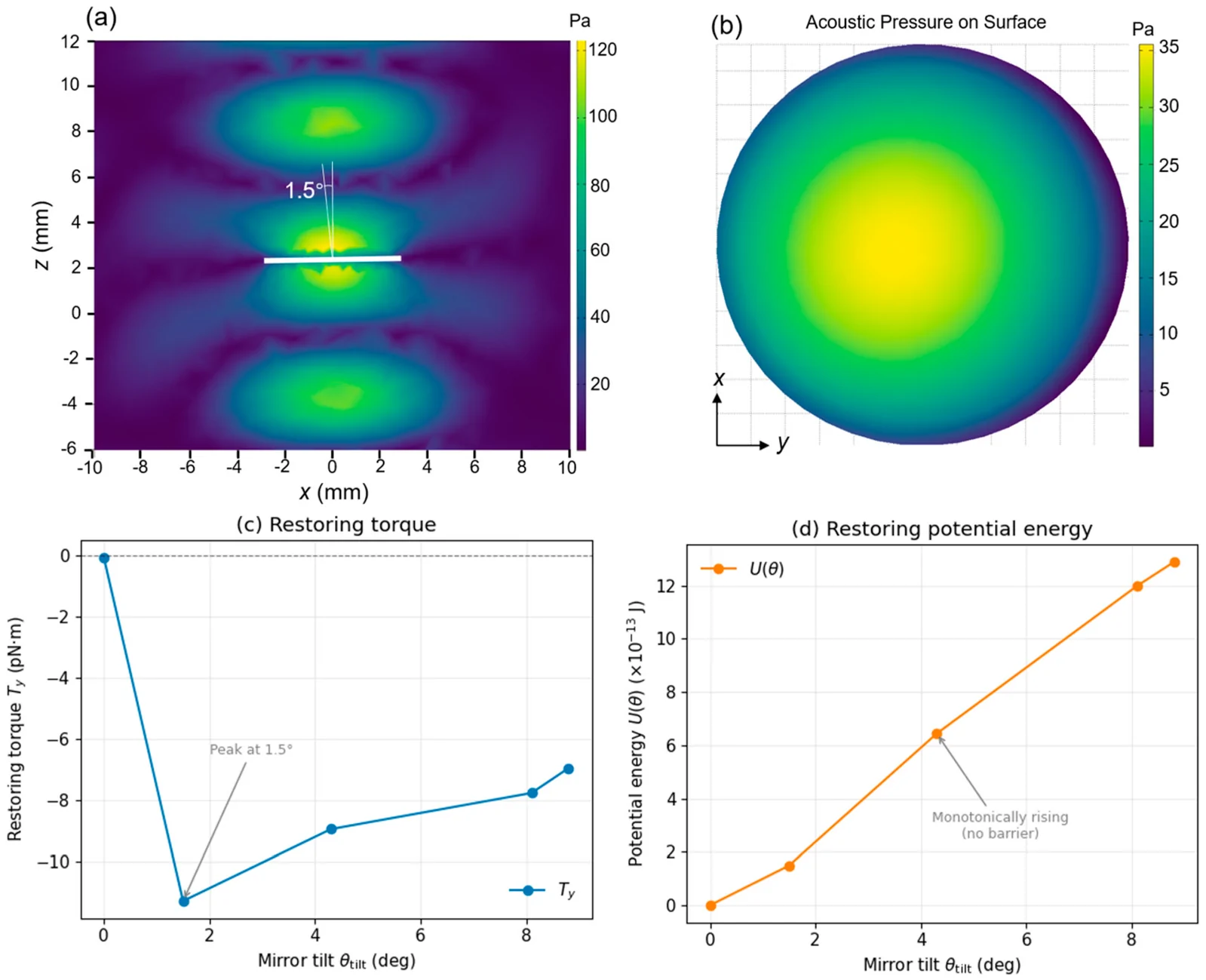
Full-wave FEM analysis of the tilt restoring behavior. (**a**) Acoustic pressure field (SPL) around the mirror tilted at 1.5°, showing tilt-induced asymmetric scattering and field distortion. (**b**) Non-uniform acoustic pressure distribution over the mirror surface at 1.5°; pressure is higher on the raised side, generating a restoring torque. (**c**) Surface-integrated restoring torque *T*_y_ versus tilt angle θ; *T*_y_ remains restoring (*T*_y_ < 0) over 0–8.8° with no sign reversal, retaining 62% of its peak at 8.8°. (**d**) Restoring potential energy *U*(*θ*) rises monotonically without a barrier, confirming rotational stability throughout the operating range.

Figure 3a shows the full-wave acoustic field around the mirror tilted at 1.5°. The tilt breaks the symmetry of the incident field, and the mirror scatters the wave asymmetrically, producing a clear distortion of the acoustic field around the tilted reflector. Figure 3b shows the resulting pressure distribution over the mirror surface, which is markedly non-uniform: the acoustic pressure on the raised side is significantly higher than on the lowered side. This pressure asymmetry is the direct physical source of the restoring torque. The surface-integrated torque *T*_y_ remains restoring (*T*_y_ < 0, opposing the tilt) over the full operating range 0–8.8°, with no sign reversal (Figure 3c):

The associated potential $U\left(\theta \right)=-\int_{0}^{\theta }T_{y}d\theta^{\prime }$ rises monotonically without a barrier maximum (Figure 3d), confirming rotational stability throughout the operating range. The torque magnitude peaks near 1.5° and then softens gradually—a reduction in torsional stiffness, not a loss of stability—retaining about 62% of its peak at 8.8°. In contrast, the lateral stiffness *k_x_* falls sharply past φ ≈ 1.0 rad and approaches zero at the steering boundary (*φ* = *π*, θ = 8.8°, Figure 2d). The rotational restoring is thus far more robust than the translational stiffness at the steering limit, confirming that the maximum stable tilt is governed by the loss of lateral confinement rather than by any loss of rotational restoring.

**Figure 4:**
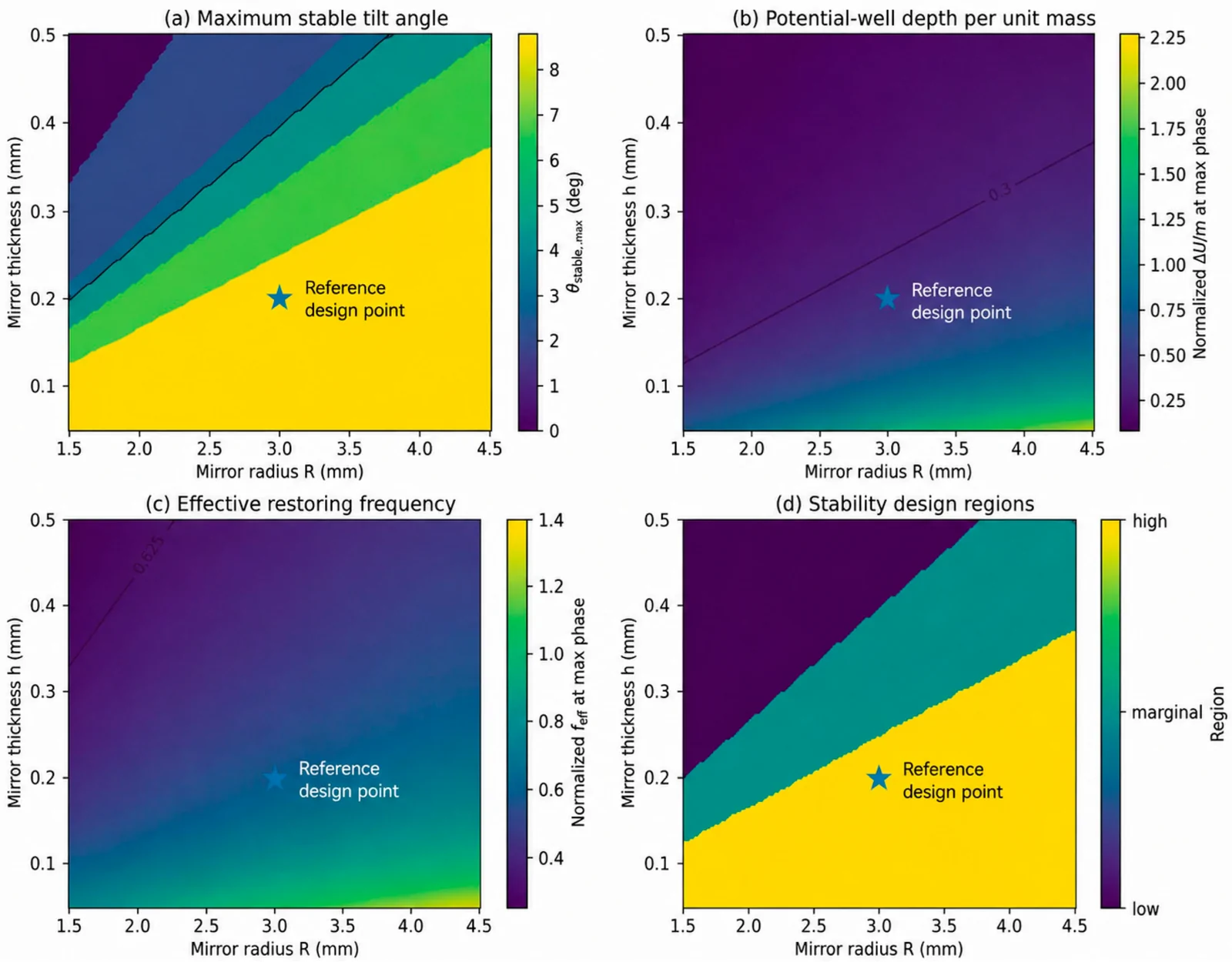
Geometric scaling effect maps and stability design zoning. Two-dimensional parametric sweep profiles mapping the influence of mirror radius *R* and thickness *h* on (**a**) the maximum stable tilt angle *θ*_stable, max_; (**b**) the specific potential well depth per unit mass Δ*U*/*m*, and (**c**) the effective restoring frequency *f_eff_*; (**d**) Synthesized engineering design map classifying the parameter space into high stability, marginal stability, and low stability regions.

To counteract the phase-gradient-induced stability degradation detailed above, the geometric design of the thin reflective plate must be optimized based on the geometry-dependent acoustic coupling and the thin-disk mass scaling (*m* ∝ *R*^2^*h*). A comprehensive two-dimensional parameter sweep was conducted by varying the mirror radius *R* from 1.5 mm to 4.5 mm and the thickness *h* from 0.1 mm to 0.5 mm. The resulting design maps evaluating the critical stability indicators are compiled in Figure 4.

The distribution of the maximum stable tilt angle *θ*_stable, max_ within the geometric parameter space reveals that the highly stable regime (where angles exceed 7°) is concentrated toward the lower-right quadrant, corresponding to large mirror radii (*R* > 3.0 mm) and small thicknesses (*h* < 0.25 mm). This trend is physically driven by the acoustic interception area: a wider mirror surface intercepts a larger portion of the acoustic wave momentum, thereby generating a higher restoring torque, whereas a minimized thickness suppresses the gravitational load and the rotational inertia of the body. The baseline configuration used in our previous experimental work (*R* = 3.0 mm, *h* = 0.2 mm) is positioned right at the boundary of this optimal zone, achieving a maximum stable tilt angle of approximately 7.5°.

The specific safety margin and dynamic stiffness are further evaluated via the unit-mass potential depth Δ*U*/*m* and the effective localized restoring frequency *f_eff_*. Both performance maps display a matching optimal topology. Because the total mass *m* scales linearly with thickness hand quadratically with radius *R*, thin-plate geometries with high aspect ratios significantly maximize the net acoustic force relative to gravity. This yields an exceptionally deep specific energy barrier and an elevated natural restoration frequency, which are essential for suppressing low-frequency environmental vibrations.

To synthesize these findings into actionable engineering guidelines, the geometric design space can be partitioned into three distinct operational zones based on a comprehensive stability index:

High Stability Region: Optimal for robust continuous beam steering, characterized by high stiffness and large angular margins.

Marginal Stability Region: Capable of maintaining static levitation but highly prone to structural escape under dynamic phase transitions or external air currents.

Low Stability Region: Structurally unviable; the mirror cannot be reliably trapped even at zero phase gradient due to insufficient acoustic radiation force relative to its self-weight.

The boundary separating these regions follows a distinct diagonal threshold. If the mirror thickness *h* is excessive or the radius *R* is insufficient, the system inevitably falls into the low stability zone. The baseline design from our previous proof-of-concept prototype sits securely within the high stability territory. This validates the empirical parameters selected in the prior experimental study and indicates that further optimization can be achieved by moving deeper into the high aspect-ratio domain (e.g., targeting *R* = 3.5 mm and *h* = 0.15 mm) to achieve even larger continuous beam-steering angles with enhanced positional rigidity.

### 2.2. Use of Generative Artificial Intelligence Tools

The Doubao generative artificial intelligence tool was used solely for non-scientific visual refinement of Figure 1a, including schematic background generation, lighting-effect enhancement, and image-resolution improvement. The scientific concepts, system architecture, physical models, and all research-related information presented in this manuscript were designed and verified by the authors. No AI tool was used to generate, modify, or interpret experimental structures, numerical data, simulation results, or scientific conclusions.

## 3. Impact of Non-Ideal Driving Conditions and Dynamic Response Limits

The static theoretical framework established in Section 2 assumes an idealized acoustic environment driven by monochromatic, perfectly tuned, and continuous harmonic waves. However, practical implementation of a phased-array beam-steering system is subject to hardware limitations, including power constraints, frequency detuning, spectral broadening, and harmonic distortion. To bridge the gap between theoretical potential and engineering application, it is imperative to quantify how these non-ideal acoustic driving conditions degrade the trap strength and subsequently evaluate the dynamic response limitations of the thin reflective plate during active angular steering.

**Figure 5:**
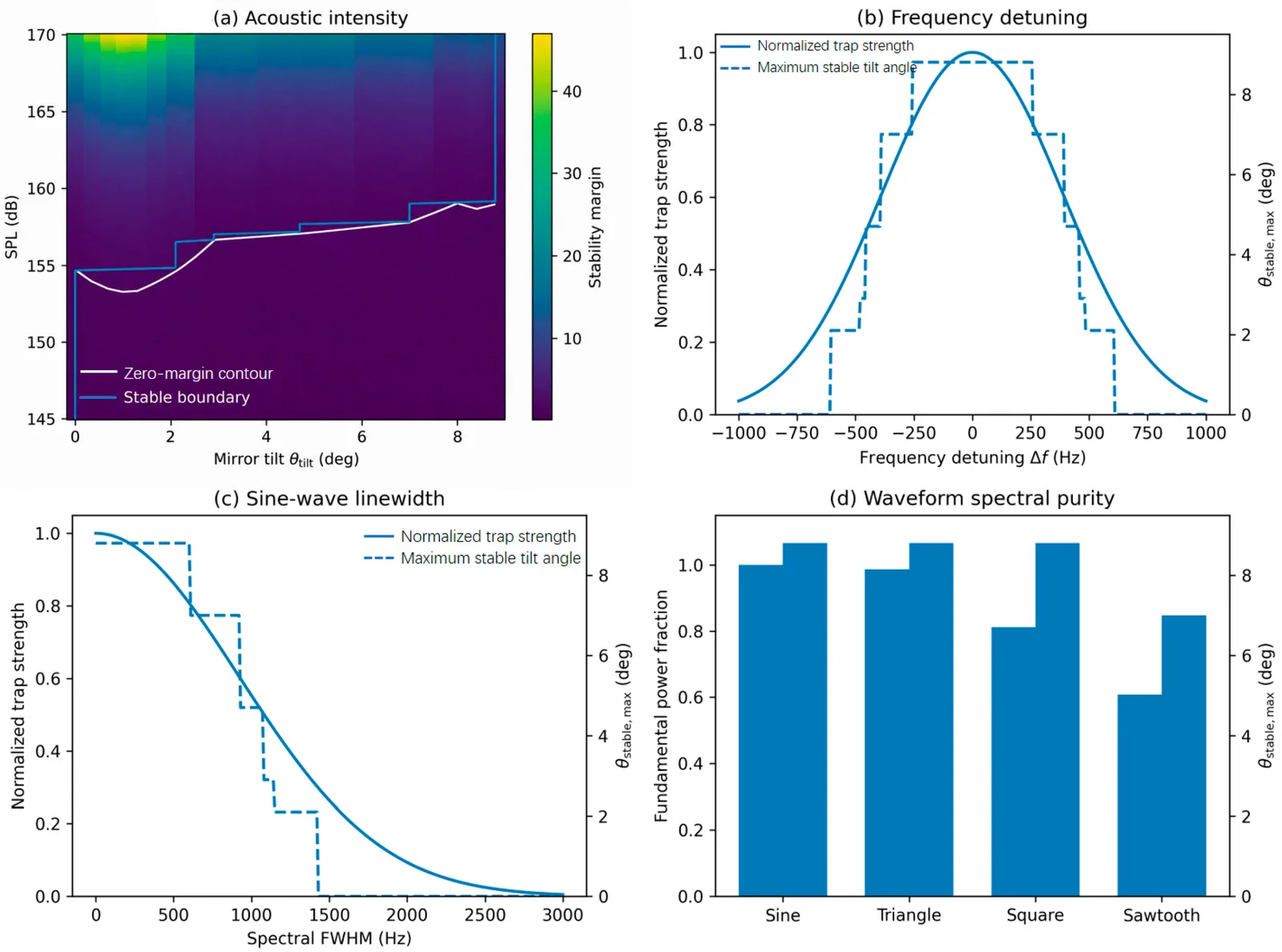
Influence of non-ideal acoustic driving conditions on levitation stability. (**a**) Stability margin versus sound pressure level (SPL) and mirror tilt angle *θ*_tilt_; the white curve denotes the zero-margin contour, and the blue stepwise curve indicates the stable boundary from discrete simulations. (**b**) Effects of frequency detuning Δ*f* on normalized trap strength and maximum stable tilt angle. (**c**) Effects of spectral linewidth (FWHM) on normalized trap strength and maximum stable tilt angle. (**d**) Fundamental power fraction and maximum stable tilt angle for sine, triangle, square, and sawtooth excitations.

The robustness of the acoustic trap relies heavily on the quality and intensity of the driving acoustic source. The degradation of levitation stability across various non-ideal acoustic parameters is systematically quantified in Figure 5.

The foundational requirement for stable levitation is sufficient acoustic energy density. As mapped in the SPL contour, the maximum achievable mirror tilt angle is heavily restricted at lower acoustic intensities. For instance, at an SPL of 155 dB, the mirror can only sustain a tilt of approximately 2.0° before the stability margin approaches zero. To reliably unlock the full wide-angle steering range up to 8.8°, the driving SPL must be maintained above a threshold under the present simulation conditions of 160 dB.

Beyond pure intensity, the spectral precision of the driving signal critically dictates the spatial coherence of the acoustic potential landscape. Frequency detuning Δ*f* from the primary array resonance causes a dramatic, Gaussian-like decay in the normalized trap strength. A detuning of just ±500 Hz halves the trap strength and severely truncates the allowable steering angle. Similarly, the spectral purity of the driving signal must be tightly controlled. As the spectral full-width at half-maximum (FWHM) broadens, the acoustic energy disperses, leading to a monotonic drop in trapping capacity. Furthermore, analysis of different waveform shapes reveals that a pure sinusoidal wave delivers the highest fundamental power fraction, yielding the maximum stable tilt. In contrast, waveforms rich in high-frequency harmonics (such as Square and Sawtooth) waste acoustic power on non-resonant modes, substantially reducing the fundamental structural rigidity of the acoustic trap.

**Figure 6:**
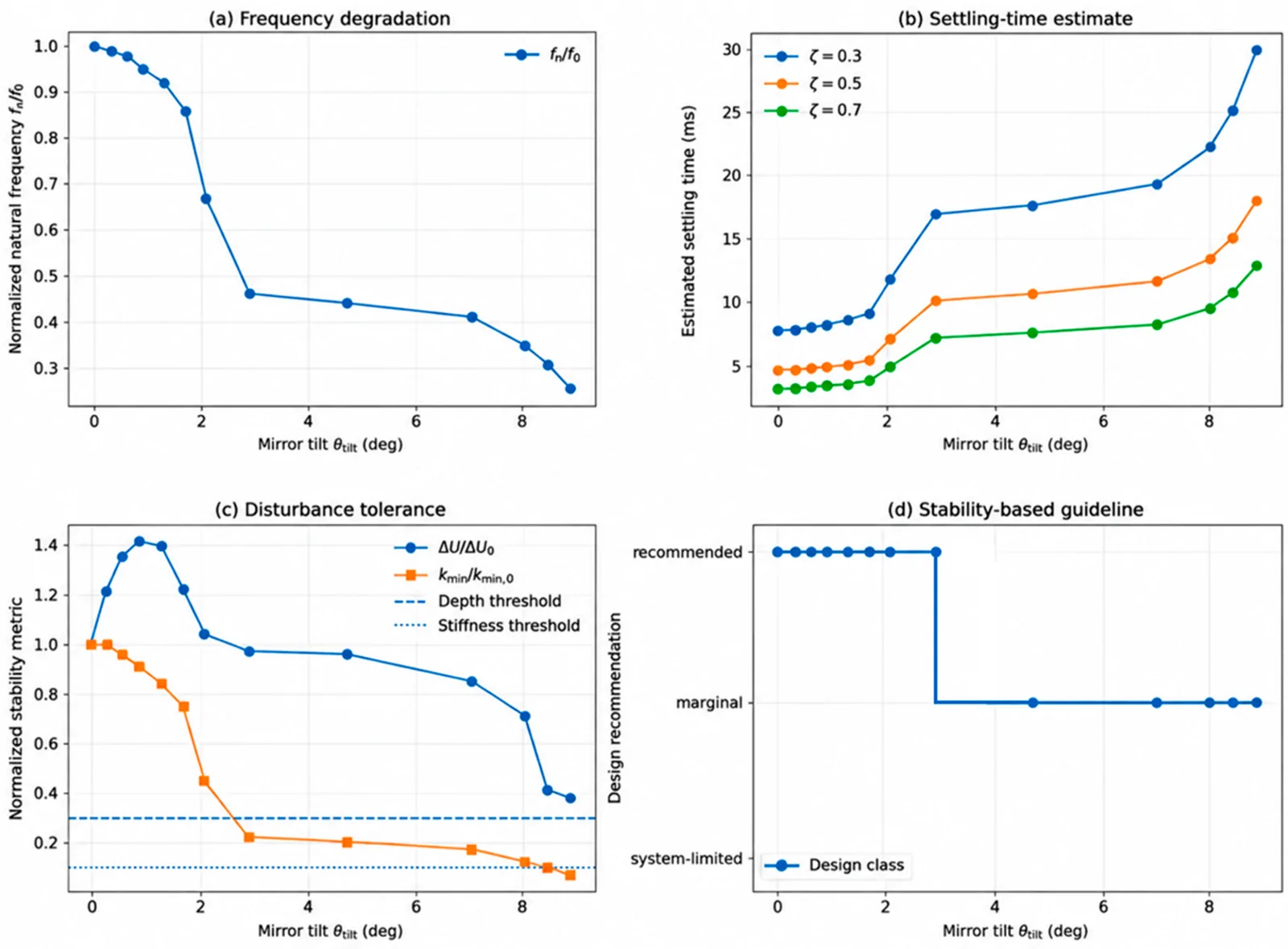
Dynamic response degradation and operational stability guidelines during active beam steering: (**a**) Normalized natural frequency *f*_n_/*f*_0_ evolution as a function of the commanded mirror tilt; (**b**) Estimated mechanical settling time required for the mirror to stabilize under different viscous damping ratios *ζ*; (**c**) Normalized disturbance tolerance evaluated via depth and stiffness thresholds; (**d**) Synthesized engineering guideline classifying the operational tilt range into recommended, marginal, and system-limited regimes.

In addition to static source constraints, active beam steering forces the thin reflective plate to continuously track a shifting acoustic potential minimum. As the mirror rotates, the severe distortion of the potential well inevitably alters the intrinsic dynamic properties of the opto-acoustic system. The transient responsiveness and stability threshold evolutions during the tilting process are detailed in Figure 6.

As the phase gradient increases to tilt the mirror, the localized structural integrity of the acoustic well softens significantly. This acoustic “softening” manifests directly as a drop in the normalized natural restoring frequency *f*_n_/*f*_0_. Initiating from a baseline lateral restoring frequency *f*_0_ of approximately 40 Hz at the unsteered 0° state, the lateral dynamic stiffness falls precipitously, dropping below half of its baseline near a tilt of 2.9°, which defines the knee of the softening curve. Beyond this knee, the frequency enters a low-stiffness plateau, decreasing monotonically to about 26% of baseline at 8.8°. Since *f*_n_ ∝ √*k*_x_, the half-frequency knee corresponds to the lateral stiffness *k*_x_ falling to roughly one-quarter of its baseline value. Consequently, this lower natural frequency fundamentally limits the system’s reaction speed. The estimated settling time map demonstrates that as the tilt angle increases, the time required for the mirror to damp out residual oscillations and stabilize at its new target angle increases drastically, particularly under low-damping conditions characteristic of ambient air (e.g., *ζ* < 0.3).

Simultaneously, the system’s tolerance to dynamic disturbances deteriorates. Tracking the normalized stability metrics reveals that while the relative potential depth Δ*U/*Δ*U*_0_ exhibits a brief initial peak before declining, the normalized localized stiffness *k*_min_/*k*_min,0_ continuously plummets, rapidly approaching the critical failure threshold at extreme angles. This indicates that stiffness degradation, rather than pure energy barrier collapse, acts as the primary failure mechanism during active steering.

By synthesizing these dynamic and static degradation metrics, a definitive operational guideline is established for the phased-array beam-steering platform. The continuous operational envelope is partitioned into three distinct classes:

Recommended Design Class (0–2.9°): bounded by the knee of the *f*_*n*_/*f*_0_ curve (Figure 6a), below which the system retains high dynamic stiffness, high natural frequency (approaching the 40 Hz baseline), and rapid settling. At this knee, *f*_*n*_/*f*_0_ falls below half its baseline (*k_x_* below one quarter), marking the practical limit of the high-stiffness regime.

Marginal Design Class (2.9–8.8°): beyond the knee, *f*_*n*_/*f*_0_ enters a low-stiffness plateau; static levitation is achievable, but the reduced lateral stiffness causes long settling times and high sensitivity to environmental jitter.

System-limited Class (beyond 8.8°): the lateral stiffness *k_x_* approaches zero at *φ* = *π* (Figure 2d), producing loss of lateral confinement and a high risk of mirror ejection.

These boundaries are consistent with the failure mechanism identified in Section 2: it is the degradation of the lateral restoring stiffness *k_x_*, rather than energy-barrier collapse, that governs the loss of stability. Full-wave finite-element simulation (Section 3) confirms that the rotational restoring torque remains robust (≈62% of peak at 8.8°) throughout this range.

## 4. Conclusions

This study has established a comprehensive theoretical and numerical simulation framework to evaluate the levitation stability and dynamic response limitations of a phased-array-driven acoustic steering thin reflective plate. By systematically bridging micro-acoustic field mechanics with rigid-body rotational dynamics, we have successfully mapped the operational boundaries of this contactless optical manipulation platform, providing actionable guidelines for high-precision opto-acoustic engineering. The primary conclusions of this work are summarized as follows:

Geometric Scaling and Structural Optimization: Based on the geometry-dependent scaling of acoustic interaction and the mass scaling of a thin disk (*m* ∝ *R*^2^*h*), we demonstrated that high-aspect-ratio, thin-plate geometries significantly maximize the net acoustic radiation force relative to gravitational loads. Two-dimensional geometric parametric sweeps confirmed that a larger mirror radius (*R* > 3.0 mm) coupled with a minimized thickness (*h* < 0.25 mm) expands the stable tilt threshold. The optimized baseline configuration (*R* = 3.5 mm, *h* = 0.15 mm) achieves a maximum theoretical mirror-tilt angle of 8.8°.

Failure Mode Identification under Phase Steering: Continuous phase-gradient modulation distorts the localized acoustic-potential landscape, inducing severe asymmetric deformation of the potential well. Our analysis reveals a critical divergence in structural failure modes: while the axial restoring stiffness *k_z_* degrades linearly, the lateral restoring stiffness *k_x_* undergoes a precipitous, rapid drop past 1.0 rad. This indicates that the primary failure mode during wide-angle beam steering is lateral slippage and escape, rather than vertical unseating.

Sensitivity to Non-Ideal Driving Conditions: The robustness of the acoustic trap is highly sensitive to hardware and excitation constraints. Under the present simulation conditions, stable operation across the full angular range requires the driving SPL to exceed approximately 160 dB. Frequency detuning Δ*f* beyond ±500 Hz halves the trap strength through a Gaussian-like decay, while finite spectral linewidth broadening monotonically erodes trapping capacity. Furthermore, pure sinusoidal excitation provides the optimal fundamental power fraction, whereas harmonic-rich waveforms (Square, Sawtooth) induce substantial stiffness degradation.

Acoustic Softening and Dynamic Operational Zoning: As the mirror tilts, the spatial disruption of the localized standing wave induces an acoustic “softening” effect. This causes the lateral restoring frequency *f*_n_ to fall to about one-quarter of its 40 Hz baseline at the largest tilt angles, triggering prolonged, underdamped mechanical ringing and low-frequency jitter in ambient air.

In summary, the theoretical models and numerical insights developed in this work provide a rigorous foundation for reducing trial-and-error optimization and guiding the design of next-generation contactless optical scanners and laser steering systems.

## Data Availability

The original contributions presented in this study are included in the article. Further inquiries can be directed to the corresponding author.
